# Mechanobiological induction of long-range contractility by diffusing biomolecules and size scaling in cell assemblies

**DOI:** 10.1038/srep27692

**Published:** 2016-06-10

**Authors:** K. Dasbiswas, E. Alster, S. A. Safran

**Affiliations:** 1 James Franck Institute and Department of Chemistry, University of Chicago, Chicago, IL 60637, USA; 2 Department of Materials and Interfaces, Weizmann Institute of Science, Rehovot 76100, Israel; 3 Department of Chemical Engineering, Northwestern University, Evanston, IL 60208, USA

## Abstract

Mechanobiological studies of cell assemblies have generally focused on cells that are, in principle, identical. Here we predict theoretically the effect on cells in culture of locally introduced biochemical signals that diffuse and locally induce cytoskeletal contractility which is initially small. In steady-state, both the concentration profile of the signaling molecule as well as the contractility profile of the cell assembly are inhomogeneous, with a characteristic length that can be of the order of the system size. The long-range nature of this state originates in the elastic interactions of contractile cells (similar to long-range “macroscopic modes” in non-living elastic inclusions) and the non-linear diffusion of the signaling molecules, here termed mechanogens. We suggest model experiments on cell assemblies on substrates that can test the theory as a prelude to its applicability in embryo development where spatial gradients of morphogens initiate cellular development.

In the 1970s Wagner and Horner^1^, motivated by the suggestion of Alefeld^2^, combined elasticity theory with statistical mechanics to predict the elastically mediated interactions of small atoms in metals. The macroscopic lattice deformations induced by these elastic inclusions^3^ are long-ranged (dipolar), and the consequent diffusion and assembly of the atoms depend on the sample shape. Similar ideas have recently been applied to living cells that adhere to an extracellular matrix (ECM)^4^. These interact through mutual, contractile deformations^5^ of the underlying matrix by forces generated by molecular motors (myosin) that act on the cytoskeleton, a network of crosslinked, filamentous biopolymers that forms the structural framework of a cell^6^. Due to acto-myosin activity^4^, the cells contract the matrix and each cell can be idealized as a contractile force dipole^5^, in analogy with inclusions in solids. However, due to the active nature of this contractility, the cell can regulate the dipole strength and symmetry and here lies an important difference between live and dead matter.

The field of mechanobiology, or “cell mechanics” to be more specific, focuses on how cells generate, sense and respond to mechanical stimuli such as forces^7^. Recent advances in this field suggest that the mechanical microenvironment of a cell, particularly its rigidity^8^,^9^, influences key aspects of cell structure and functionality. This demonstrates the importance of elastic interactions that can be mediated by deformations of the cytoskeleton within a cell or of the substrate or extra-cellular matrix between cells. These ideas have been used to explain the experimentally observed dependence of organization of the cytoskeleton on substrate stiffness^4^,^10^,^11^,^12^. In addition to the role of the mechanical environment on physico-chemical properties such as the organization of the cytoskeleon or cell-cell forces^13^, measurements of the role of mechanics in the differentiation^14^ and development of the cytoskeleton of stem cells^10^,^15^ and in gene expression in mature cells^16^ have demonstrated that biological function can be strongly modulated by the sensitivity and response of cells to mechanical cues. While the mechanobiology community has typically treated assemblies of isolated adherent cells that are in principle homogeneously contractile, this is in fact not always the case, as in cell monolayers important in motility and wound healing assays^17^. The cells at the periphery of the monolayer are in principle different from those closer to the center^18^. Such assemblies are of course subject to internal mechanical forces. The results presented in this paper suggest that these mechanical forces that originate in contractility can be coupled to biochemical diffusion that can further influence the contractility of the monolayer, an effect that, though plausible, is yet to be investigated in a mechanobiological context. In addition, such effects may be relevant to pattern formation in tissue development. All of these motivate our investigation of the role of gradients of biochemical signaling molecules and their feedback with cellular contractility. Inspired by this idea from developmental biology, but considering cells in culture as a first step, we denote such molecules that induce cytoskeletal contractility in a concentration-dependent manner as “mechanogens” (analogous to “morphogens” in embryo development^19^).

In addition to their role in the structural organization of the cellular cytoskeleton of isolated cells, elastic interactions between cells provides an additional strategy for long-ranged inter-cellular signaling, which can be much faster than the diffusion of chemical signals^20^,^21^. The idea that mechanics, via the forces^22^,^23^ and flows^24^,^25^ generated by active cellular processes, interacts with chemical signaling to regulate various aspects of development has led some authors to suggest a “mechanochemical basis” of morphogenesis^26^,^27^,^28^. While the crucial role of physical forces and dynamics in aspects of development was historically appreciated^29^, it has only recently begun to be quantified^26^ in specific model systems. In contrast with prior mechanochemical models that consider either the hydrodynamic flow of cytoskeletal elements^25^ or the hydrostatic mechanical pressure^30^ created passively through tissue growth, and their feedback on morphogen dynamics, we focus on elastic interactions in adherent (non-motile) cells. By treating the feedback between biochemical signaling and the long-range shear strains actively induced by cytoskeletal contractility – we derive a novel principle for the scaling of the biochemical gradient with size of the cellular assembly. We emphasize that in this paper, as a first step, we focus on model systems of adherent cells cultured on synthetic substrates typically studied in mechanobiology^8^,^9^. These cells remain adhered to the substrate for long time scales of tens of minutes^31^ to hours on patterned substrates with regions of strong adhesion (private communication with A. D. Bershadsky). This gives rise to large-scale cytoskeletal structures and adhesion sites^32^ whose disassembly occurs over tens of minutes, unlike motile or cytokinetic cells whose cytoskeleton undergoes rapid turnover and is more fluid-like^24^. Our theoretical focus on elastic interactions in strongly adhered cells with stable large-scale cytoskeletal structures that act as sources of active stress, is thus complementary to the hydrodynamic description of the cytoskeleton as an active fluid^33^ relevant for motile cells. While the mechanobiology experiments based on our predictions are appropriate to cell cultures, the phenomena may eventually lead researchers to extend the physical experiments to understand how contractility gradients are established in developing tissue, where viscoelastic effects do become more important^34^,^35^. The immediate goal of our paper is to quantitatively predict in a self-consistent manner, the molecular concentration and contractility profiles that vary in a cell assembly for which contractility is initially introduced in a local region, analogous to the situation for morphogens. We consider well-adhered cells cultured on substrates as a tractable and experimentally testable system that eventually can be extended to treat the *in-vivo* case of cells that are stably adhered to the extra-cellular matrix. This approach allows us to decouple the molecular diffusion and mechanics from any possible flow of the cells themselves. We show how both these profiles can generically be long-ranged, scaling with the system size over a robust range of parameter values.

This scaling behavior is inspired by the observation that tissue patterning often scales with the size of the embryo as dramatically illustrated in the classic experiments by Spemann and Mangold^36^ who showed that an amphibian embryo when cut into half in the blastula stage could still grow into two well-proportioned adult individuals^37^. Had the morphogen gradient not scaled with size, one would not expect the “distal ends” (far from the morphogen source) of both larger and smaller embryos to show nearly identical developmental patterns. Size scaling of the morphogen gradients has also been seen in the context of the growing fly-wing^38^ and the anterior-posterior patterning of the fly body^39^. Mechanisms based on the coupled reaction and diffusion of morphogens and inhibitors have been proposed to explain scaling of the morphogen gradient with embryo size^40^, such as in amphibians^41^,^42^. Our mechanochemical model for cellular contractility is different in spirit from such reaction-diffusion mechanisms introduced by Turing^43^, where the chemical interactions between two or more diffusing species is responsible for pattern formation. Instead, we focus upon the interplay of one *mechanical* (*i.e.*, physical) and one chemical factor and treat both at scales where the molecular details are unimportant. The fact that biological systems are inherently mechanochemical begs the question if long-ranged chemical and mechanical gradients in an assembly of cells where mechanogens trigger contractility in an inhomogeneous manner could be realized through a mechanochemical feedback. We show that this is indeed theoretically plausible and describe a possible mechanism which biological systems may have evolved to exploit.

## Mechanodiffusive model and analysis

**Figure 1:**
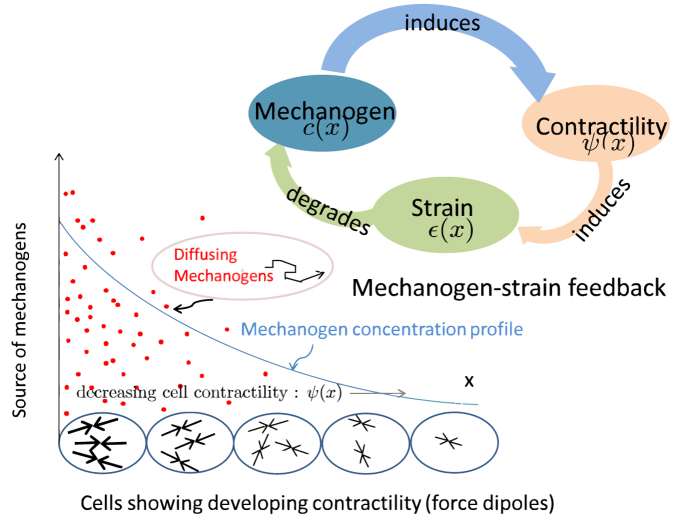
Schematic illustration of our model for cell-mechanogen coupling showing an array of cells representing an initially non-contractile cell-assembly subject to a concentration gradient of contractility-inducing, diffusing molecules (mechanogens), shown as red dots, which are introduced at a localized source, here confined to the *x* = 0 plane. The diffusion and the degradation (or capture) of these molecules by the cells compete to result in a steady-state gradient of both the contractility and the mechanogen concentration. The cells to the left are exposed to a higher local mechanogen concentration. Hence those cells are more contractile and have more numerous and/or more highly contractile acto-myosin rich regions, represented in our model as coarse-grained “force dipoles”^4^. The inset shows how our model allows for feedback between the cell contractility and the concentration of the mechanogens through the strains induced in the elastic medium. While the mechanogens induce cell contractility, the receptor-mediated uptake or degradation of these mechanogens can be, in turn, promoted by cell strain in the negative-feedback scenario we consider here. In the context of development, the varying contractility of the cells in an array can distinguish different organs in an embryo; however, the theory and experiments discussed here focus on their application in the mechanobiology of cells in culture.

Morphogens are soluble proteins that regulate gene expression in a concentration-dependent manner and play a crucial role in the pattern formation processes that lead to the development of a complex, differentiated organism from a single cell^19^. In the simplest case, the morphogens are produced at local sources within a developing embryo and diffuse through the extracellular fluid to form a concentration gradient. The genetic changes they trigger facilitate cellular development including presumably cytoskeletal changes that are manifested as a gradient in cell shape^44^,^45^. It is reasonable to think that this can be related to a gradient of cell contractility. Inspired by this idea, we consider mechanogens that induce cytoskeletal contractility in proportion to their concentration whose spatial dependence we denote by *c*(x), and whose activity can in turn be affected by contractility (see Fig. 1). We focus on cell assemblies in culture and predict how diffusion gradients of the mechanogens and the contractility gradients can couple to give inhomogeneously contractile cell arrays whose characteristic gradients can scale with the system size.

Cells are known to mechanically interact with each other over long distances^13^ through active, contractile forces which are propagated either through deformations of the underlying ECM/substrate in a sparse cell culture or direct transmission of the contractile forces from one cell to another via their mutual adhesive contacts in a dense cellular assembly. An adherent, non-motile cell whose cytoskeleton does not undergo rapid re-organization remains contractile and induces elastic deformations over relatively long time scales^4^,^35^. The contractile regions within cells whose contractility is first developing^10^ (as well as in cells with well-established stress fibers) can be represented as a distribution of active “force dipoles”, tensorial quantities of the form *p*_*ij*_(x) used to describe the typical contractile “pinching” force pattern produced in the cellular cytoskeleton^4^,^5^. We assume here that cells subject to a higher concentration of mechanogens are more contractile and to a first approximation, mechanogens induce cellular contractility but not yet its orientation within the cell. Each cell can be then represented as an isotropic force dipole (with equal components in all directions), corresponding to a force dipole density of the form *p*_*ij*_(x) = *p*(x)*δ*_*ij*_, where *p* represents the strength of a force dipole, and is a measure of the radial contractile forces applied by myosin motors in the cellular cytoskeleton. We denote by *ψ*(x) the cell’s contractility normalized to its maximum value: *ψ*(*x*) = *p*(*x*)/*p*_*max*_, so that the value of *ψ* is bounded between *ψ* = 0 for a non-contractile cell and *ψ* = 1 representing a fully contractile cell.

The local contractility depends linearly on the local effective mechanogen concentration near the cell surface in our model, while the strain induced in the elastic medium (cells +ECM) has a local compressive contribution from the cell at that particular position as well as a contribution from all the *other* contractile cells (force dipoles) as,


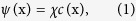



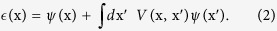


Here, 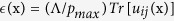 is the normalized trace of the local strain tensor at position x, Λ an appropriate elastic modulus (derived in the Methods), and *V*(x, x′) (which has units of inverse volume) accounts for the generally, long-ranged mechanical interactions of the contractile cells so that a cell at a position x′ affects a cell at position x arbitrarily far away, through the strain it induces in the surrounding elastic medium there. Compressive strains and contractile dipoles are both, by convention, negative, such that the normalized local strain we define here is positive, 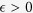, for a local compression. The cellular contractility is expressed by their dimensionless (since *χ* has units of volume and c(x) is the concentration of mechanogen) force dipole density, *ψ*(x). Eq. (1) expresses the fact that the mechanogen induces cell contractility in a local manner via the “susceptibility” *χ*. An additional possibility, that the local contractility is also actively changed by the cell in response to the local strain induced by the other contractile cells, such as through mechanically transduced myosin regulation^46^, is addressed in the SI. This gives results identical to the situation presented here, depending on the mechanical boundary conditions. Since mechanical forces are transmitted very fast^20^, the cellular assembly can be considered to be in local mechanical equilibrium even if the mechanogens are both chemically produced and “degraded”, *i.e.*, removed from the extracellular fluid, and hence, never in chemical equilibrium.

We close the mechanogen-contractility-strain feedback loop by considering two limiting cases where the rate of degradation of mechanogens is either (i) purely biochemical^47^,^48^ and independent of the mechanical state of the cell, or (ii) depends linearly on the local strain. In the former case, the mechanogens can be degraded both in the extracellular fluid by chemical reactions with other molecules and intracellularly after uptake by the cells^47^,^48^. In case (ii), there is strong feedback between the mechanogen degradation and the local strain, applicable, for example, when uptake of the mechanogens from the solution involves transport across the cell membrane, such as by receptor-mediated endocytosis, the rate of which is affected by the membrane tension^49^. The strong feedback of degradation and contractility is appropriate when purely biochemical degradation of type (i) is very slow. Since rates of degradation are exponentially sensitive to the relevant binding energies, the strain-enhanced binding of mechanogens to the cytoskeleton may be very large. The spatially inhomogeneous mechanogen concentration profile is at steady state when the overall rates of its production and degradation balance^40^ and can then be described by the diffusion-degradation equation^38^,^40^


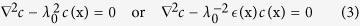


for cases (i) and (ii) respectively. Here, the molecular diffusion constant is *D* and *λ*_0_ is a “molecular” or cellular length scale (independent of system size) associated with mechanogen degradation: the inverse time, 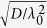, is the rate of degradation of effective mechanogens in case (i) and that for a maximally contractile cell in case (ii). The solution for *c*(x) in case (i) decays exponentially as exp(−*x*/*λ*_0_) in one dimension (d = 1) and more generally as exp(−*r*/*λ*_0_)/*r*^(*d*−1)/2^ for *d* = 1, 2, 3. While *λ*_0_ is determined by the local biochemistry and is thus independent of the size of the cellular array (unless specific biochemical and boundary conditions are satisfied as in ref. 41), the contractility profile, even in case (i), can still be long-ranged as discussed later on. Case (ii) is the more interesting situation of strong degradation-contractility coupling through which the system-size dependence of the strain can result in system-size dependence of the effective degradation length, 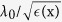. It is precisely because different cells develop different contractilities that there is a spatial gradient of the strain field 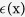 and the effective degradation length scale can be much larger than the molecular length scale *λ*_0_.

We consider first the elastic interactions between two contractile cells as two force dipoles embedded in or on an elastic medium given by the interaction energy, *V*(x, x′). We show in the Methods section that this scales as the inverse of the system volume, and is to a first approximation, independent of the separation between the dipoles: *V*(x, x′) = *V*_0_/*L*^*d*^, where *d* is the dimension of the system, and *V*_0_ is a dimensionless constant that is positive(negative) for free(clamped) boundaries^1^ for *d* = 2 and 3. Such a size-dependent elastic interaction is intuitively expected because a pair of isotropic force dipoles embedded in an infinite elastic medium do not interact elastically^50^; however, in a finite medium they do interact through the boundaries^51^ by inducing “image dipoles”. Such predominantly boundary-dependent interactions also arise from a continuous distribution of force dipoles, *ψ*(x), which corresponds to a surface distribution of forces (the forces cancel out in the bulk in a continuum approximation). We derive the form for such elastic interactions explicitly for a spherically symmetric distribution of force dipoles in the Methods section, but as we explain there, the non-spherical modes of interaction are also long-ranged and scale inversely as system volume^1^. Elastic interactions between dipoles in 1D can be derived by considering a series of springs (see SI) of total length *L* which gives rise to an interaction *V*(*x*, *x*′) = *V*_0_/*L* where *V*_0_ < 0 if the springs are clamped at either end, and *V*_0_ = 0 for free boundaries.

The cell located at x induces a compressive (negative) strain locally because it is contractile, whereas the dipoles located elsewhere either stretch or compress the matrix at position x depending on whether the boundaries are clamped (zero displacement) or free (zero strain). The mechanical interaction energy of a force dipole with all the others is given by the product of the local dipole and local strain (due to the other dipoles)^51^. This interaction is attractive (negative sign) for the case of clamped boundaries, appropriate for a spherical shell of cells, which is inspired by the situation in development of a blastula^52^ that is attached to a rigid surface *e.g.*, an eggshell or placental wall; in a cell culture assembly, this can be achieved experimentally by holding the matrix fixed at its outer boundary. For a system with free boundaries, inspired by a blastula with fluid yolk around it in a developmental context, the interaction is repulsive in two or three dimensions; in one dimension, the dipoles do not interact. We rewrite the expression for strain in Eq. (2) using the form of long-ranged interactions stated above as,


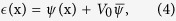


where 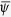 and 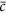 are the volume-average of the contractility and concentration respectively over the entire system, *e.g.*, 
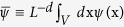
. In the situation in which the degradation is very weakly coupled to contractility, case (i), the concentration gradient is short-ranged and system size independent (unless it is coupled to another diffusible species which can make the gradient long-ranged^40^). However, due to the long-range elastic interactions, the strain, while larger near the source of mechanogens still has some residual dependence on the size of the cell assembly:


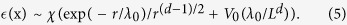


The case of strong degradation-contractility feedback gives more interesting predictions as we now explain. Using Eq. (4) and the linear relationship between contractility and local mechanogen concentration, Eq. (1), the strain is eliminated in favour of the mechanogen concentration. Eq. (3) can then be written as a function of the mechanogen concentration alone


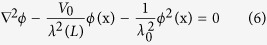


where we define a dimensionless mechanogen concentration *ϕ*(x) ≡ *χc*(x), *V*_0_ is positive (negative) for free (clamped) boundary conditions, and,


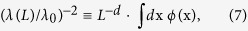


is defined as an effective decay-length that depends in a self-consistent manner on the mechanogen concentration and the size of the system. Eq. (6) is a steady state diffusion equation with a nonlinear degradation term and a linear term whose sign varies depending on the mechanical boundary conditions. In the absence of the nonlinear term and for the case *V*_0_ > 0, Eq. (6) predicts an exponentially decaying concentration profile with a decay length *λ* defined in Eq. (7). Since this decay length depends on the system-averaged concentration 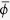, Eq. (6) must be solved self-consistently to determine 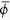 which then predicts how *λ* depends on the system size; we henceforth write *λ*(*L*) (or *λ*(*R*) in two or three dimensions) to remind us of this.

We consider a constant mechanogen flux *J* generated by a localized “source” of size *a* (much smaller than any other length scale),


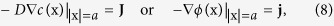


which in one dimension is located in the region −*a* ≤ *x* ≤ *a*. In two or three dimensions (radially or spherically symmetric), the mechanogen source is in the region *r* ≤ *a* and the boundary condition refers to the gradient of the concentration at *r* = *a*. Henceforth, to eliminate multiple molecular parameters, we choose *a* = *λ*_0_; the resulting theory is easily generalized. Since the cell assembly is finite (0 ≤ *x* ≤ *L* in 1D), confinement of mechanogens to the tissue implies *dc*/*dx*|_*x*=*L*_ = 0. For ease of analysis, we consider assemblies where *L* ≫ *λ*, *i.e.* the assembly is effectively infinite; this allows us to use natural boundary conditions where the concentration decays exponentially as *x* or *r* tends to infinity. The self-consistent solution of Eq. (6) predicts the dependence of the decay length *λ* on the system size.

**Figure 2:**
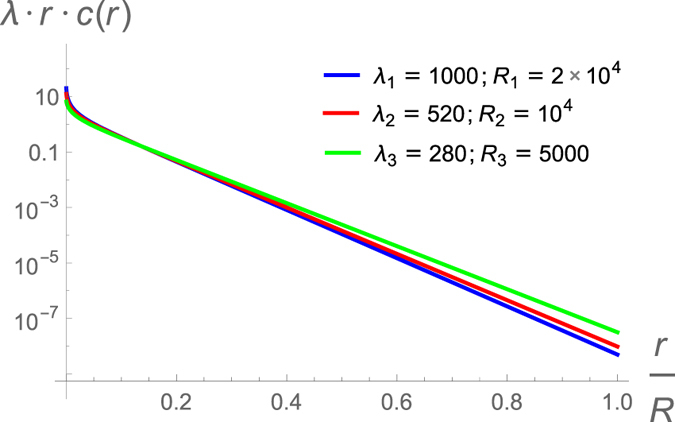
Self-consistent solutions in 3D vs. radial coordinate rescaled with system size, *R*, showing approximate scaling. We solve the diffusion-degradation equation in 3D self-consistently, numerically for three different system sizes *R*_1_ = 20,000*a* (blue), *R*_2_ = 10,000*a* (red) and *R*_3_ = 5000*a* (green)and find the corresponding decay lengths *λ*(*R*) = 1000*a*, 520*a* and 280*a* respectively from the self-consistency condition 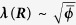 stated in Eq. (7). The solutions are multiplied by the product of the radial coordinate *r* and the corresponding *λ*(*R*), and plotted vs. *r*/*R* on a semilog scale to test the expected exponential behavior of the solution far from the source: 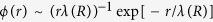, which is the “outer solution” of Eq. (6) in 3D (see approximate analysis in the Methods section). The numerical values of the decay lengths do not exactly scale with system size because the approximate analysis presented there is only correct in the asymptotic limit where *R* → ∞ so that in practice, the numerical solutions show crossover contributions from both regions 2 and 3.

Near the source of flux (the “inner region”), the concentration *ϕ*(*x*) is relatively large and the quadratic degradation term in Eq. (6) dominates the linear term resulting in a 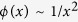 power law in this region. Sufficiently far from the source (the “outer region”), the linear term in Eq. (6) dominates and the mechanogen concentration decays exponentially- to zero for *V*_0_ > 0, and towards a small but finite value, *ϕ*_*b*_, for *V*_0_ < 0. Matching the inner and outer solutions and imposing the self-consistency condition of Eq. (6), determines the length scale of the exponential decay in the outer region. We obtain 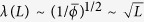 for a 1D geometry of cells on a ring, 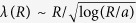 for a 2D disc geometry, and 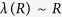 for a 3D spherical geometry (derived in the Methods section). The profile can be said to “scale” with the system size, at least in an approximate sense, if the length scale characterizing the decay of the profile, here defined as *λ*(*L*), is proportional to the system size, *L*. We see that in 1D, this scaling does not occur even though the mechanogen concentration profile is long-ranged, decaying with a length scale proportional to 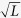. In two or three dimensions, we do obtain scaling (up to logarithmic corrections in 2D) by the arguments presented here (verified numerically and shown here for 3D in Fig. 2).

The scaling of the mechanogen and contractility profiles with the system size applies for large values of the localized flux, *J*. For small values of the flux, in 2D and 3D, the problem is linear, the quadratic term in Eq. (6) can be neglected, the concentration profile lacks the power law inner region and we predict 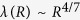 and 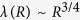 respectively. The mechanogen profile can be solved exactly in a large 1D system (see the Methods section, Eq. (15)). For *x*_0_ ≪ *λ*(*L*) there is a separation of length scales and *λ*(*L*) ~ *L*^1/2^ as discussed above. For *x*_0_ ~ *λ*(*L*) there is no separation of length scales, the profile still decays as Eq. (15)) indicates. In the limit of 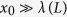, the problem linearizes and we predict 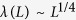. These results show that the long-ranged nature of these profiles is reduced when the source flux is reduced *i.e.* the length scale associated with the flux is no longer well-separated from the decay length.

## Discussion

Our model and its predictions depend on: (a) the generic, long-ranged nature of elastic interactions between cells as expressed in the force balance Eq. (2); (b) contractility in a cell being locally induced by the mechanogen (this is our definition of a “mechanogen”); and (c) for case (ii) of strong-feedback, the dependence of the degradation of the mechanogen on the cell strain. In the following, we focus on the predictions for case (ii), although the experiments we suggest should be tested to see if the weak coupling situation (case (i) where the mechanogen concentration decays on a molecular or single-cell scale, while the strain has a small residual dependence on the system size) or the strong-coupling case (ii), are applicable. The latter predicts that the elastic interactions of the contractile cytoskeleton of developing cells give rise to the scaling of the mechanogen concentration gradient with the size of the cellular assembly in two and three dimensions; for a one dimensional line or ring of cells, the concentration gradient decays with a length scale proportional to the square root of the system size. The dependence of decay length on system size is an outcome of the long-ranged nature of elastic interactions between contractile cells which occur through the boundaries of the finite assembly of cells, and are naturally system size-dependent. This very general feature of cell mechanics is likely to remain true in any model assembly of cells in culture, irrespective of the details of molecular interactions and signaling pathways. These results depend only on the fact that there is a region where the degradation is quadratic in concentration (which is a result of the simple bilinear coupling of contractility to mechanogen degradation) and a linear region. It is the matching of the solutions in these two regions and the self-consistency condition that give the scaling relations discussed here. We note that the time scale at which the steady-state situation can be reached depends on the two time scales in the problem: the time to diffuse across a characteristic length scale of the concentration gradient, *λ*, given by *λ*^2^/*D*, and the characteristic timescale of degradation; which both scale as *L*^2^/*D* in the case where the decay length *λ* scales as the system size (and therefore implies a few minutes to reach steady state for tissue of typical size).

The generic nature of these results should first be explored experimentally in mechanobiology experiments in cell culture. This may be of intrinsic interest to cell culture researchers that focus on cell motility and wound healing as we explained in the Introduction. However, their general nature suggests that they may be relevant to biological development, even if there is no direct evidence yet of specific morphogens that stimulate the development of cellular (cytoskeletal) contractility (however, there is indirect evidence through cell shape changes^44^,^45^, as mentioned earlier). In cell culture, *in vitro*, the nature of the scaling of the mechanogen and contractility profiles can be measured on synthetic, deformable substrates^8^ subject to mechanogens that are added at a local source located in a small region of the cellular assembly. The experiments would simultaneously measure the development of cell contractility, for example, by traction force microscopy, and the concentration profile of the chemical species, for example, by fluorescence imaging. The dependence of the chemical concentration profile on the mechanical environment can be tested by varying the stiffness of the underlying synthetic substrate or by varying the mechanical boundary conditions. There is also the possibility that more contractile cells may themselves secrete the mechanogens that could then diffuse and induce contractility in the other, initially non-contractile cells of the assembly, thus mimicking the direct injection of mechanogens in a localized region. This should be explored experimentally by inducing higher contractility in a local region, for example, by plating a few cells of the assembly on a stiffer substrate compared to the rest.

In addition to the degradation-strain feedback considered here, other kinds of mechanodiffusive couplings may also lead to scaled concentration gradients because of the ubiquitous long-ranged elastic interactions between contractile cells (see example in SI). The scaling results are robust to the mechanical boundary conditions and lead to the same size scaling of the decay length characterizing mechanogen concentration for both free and clamped boundaries. While in this work, we considered only the simplest, spherically symmetric contractility and concentration profiles, the ideas can be extended to non-spherically symmetric pattern formation – of spatial profiles of both mechanogen concentration and cellular contractility, which may be induced even for a spherically symmetric source flux through the nonlinear terms in Eq. (6). Our mechanochemical model for cellular contractility is different in spirit from reaction-diffusion mechanisms introduced by Turing, where the chemical interactions between two or more diffusing species^43^ are responsible for pattern formation. Instead, we focus upon the interplay of one *mechanical* (*i.e.*, physical) and one chemical factor and treat both at scales where the molecular details are unimportant. This is in contrast with the more biochemically specific mechanism of scaling involving (non-linear) threshold effects of at least two diffusing and reacting species suggested by Barkai *et al*.^41^ in the context of genetic (as opposed to contractility) development. To our knowledge, the theory presented here is a first step in the understanding and measurement of mechanics-based scaling of contractility-inducing chemical gradients, inspired by development, which may open up new horizons in mechanobiology.

## Methods

### Mechanical equilibrium equation

The force density corresponding to a distribution of dipoles, *p*_*ij*_(x) is given by^51^,


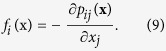


while the corresponding elastic equilibrium condition can be expressed as^53^,


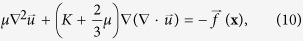


where 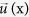 is the local displacement field created by the deforming forces from the dipoles. For a spherically symmetric distribution of dipoles in 3D, Eq. (10) for the radial displacement *u*(*r*) reduces to,


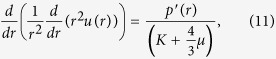


The trace of the strain (in radial coordinates) defined as *Tr*(*u*) ≡ *du*/*dr* + 2*u*/*r*, for clamped and fixed boundaries of the spherical medium is then given by,


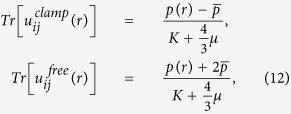


respectively. A more detailed derivation is provided in the SI.

### Scaling of the mechanogen concentration profile in 1D

We first consider the simplest possible geometry of a ring of cells that can be mapped to a one-dimensional line with periodic boundary conditions. In this mapping, the region −*a* ≤ *x* ≤ *a* contains the source of mechanogens. The mechanics of this system can either be solved using one-dimensional continuum elasticity or can be represented by a series of springs with force pairs acting on them. We show in the SI that the resulting interaction between the dipoles is independent of the distance of separation and is attractive when the springs at the boundaries are held fixed: *V*(*x*, *x*′) = *V*_0_/*L* with *V*_0_ < 0. In 1D, it is only this case with “clamped” boundary conditions that is interesting because free boundary conditions do not result in any interaction between the dipoles.

The general steady state diffusion-degradation relation, Eq. (6), can be written for *V*_0_ < 0 in a 1D system as,


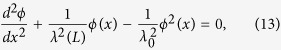


where 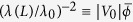 is a redefinition of the decay length. Near the source at *x* = *a* where 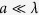 (which we term the “inner region”), the concentration *ϕ*(*x*) is large (the concentration decreases monotonically away from *x* = 0 because an incoming flux of mechanogens is maintained only at this end) and the quadratic degradation term in Eq. (6) dominates the linear term whose coefficient *λ*^−2^(*L*) decreases strongly with increasing system size, *L*, as our self-consistent calculation shows. This purely nonlinear decay has an approximate power law behavior 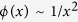. Sufficiently far from the source, (which we term the “outer region”), the concentration of mechanogens decays exponentially towards a small but finite value, *ϕ*_*b*_. This is because the (negative) term quadratic in *ϕ*(*x*) in Eq. (13) must dominate the positive, linear term if the system is to maintain a steady state. Physically, there can only be effective degradation and no production of the mechanogens locally. The solution in the outer region then behaves as 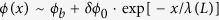, and a self-consistent determination gives: 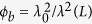. The inner and outer solutions described in the approximate analysis here are matched at around *x* ~ *λ*, and this holds when there is a clear separation of length scales: *a* = *λ*_0_ ≪ *λ* ≪ *L*. We can then use the analysis above and the self-consistency condition of Eq. (7) to determine the decay length:


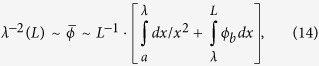


to obtain 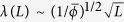, where have used that 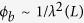. Eq. (13) for the mechanogen concentration can actually be solved exactly analytically for large systems as


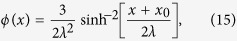


where *x*_0_ is a length scale associated with the source flux *j* related inversely to it through the boundary condition for source flux, *dϕ*/*dx*(*x* = 0) = −*j*. The large flux limit discussed above corresponds to the case where *x*_0_ ≪ *λ* (a separation of length scales).

### Scaling for 3D spherical geometry

We now predict the mechanogen concentration profile for a spherical assembly of cells with the source located at its center 0 < *r* < *a*, at which a a flux of mechanogens is introduced. The flux is isotropic: −∂*ϕ*/∂*r*|_*r*=*a*_ = *j*. We focus on the case of a spherically symmetric mechanogen concentration profile with stress-free boundaries which is the solution of:


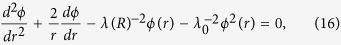


which is the specifically 3D version of the general Eq. (6) stated above for *V*_0_ > 0 and a redefined decay length, 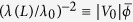. Since we analyzed a clamped boundary condition in 1D (the only simple, physically reasonable boundary condition in 1D), we analyze in detail the free boundary condition for the 3D case. The arguments for a 3D clamped case proceed very similarly to that for the 1D clamped situation in which the mechanogen concentration decays exponentially to a finite value far from the source. The scaling of the exponential decay is the same, however, for the two boundary conditions. This is because the interactions scale inversely as system size, *V*(*r*, *r*′) ~ *R*^−3^ in both cases, although they differ in the sign (and magnitude) of the pre-factor.

Since Eq. (16) does not have an exact analytic solution, we address it here with approximate analytic arguments supported by numeric solutions. One can approximate the solution by closed form expressions in different regions as we now show. It is important to identify the essential length scales in the problem: the finite size *a* of the source of particles, the length scale *r*_0_ which is related to the flux of particles andcorresponds to the region over which the source flux has a strong effect on the concentration profile, the decay length of the steady-state mechanogen profile in the far-field, *λ*(*R*), and the size of the system, in this case the radius of the spherical assembly of cells, *R*. Our approximate analysis is valid when these length scales are all well-separated, *a* ≪ *r*_0_ ≪ *λ* ≪ *R* which is applicable only if the source of mechanogens is much smaller than the embryo size. The concentration gradients of interest are steep enough close to the mechanogen source and in this region the non-linear term may dominate. For this to be relevant the flux cannot be too small, *i.e.*, *r*_0_ cannot be too large and thus, must be significantly smaller than *λ* which characterizes the region of exponential decay. When the flux at *r* = *a* is small, the nonlinear term is not relevant and the mechanogen profile is purely exponential. In this case, the concentration profile goes like *ϕ*(*x*) = *ja*^2^exp[−(*r* − *a*)/*λ*(*R*)]/*r*. This changes the predicted size-dependence as mentioned in the main text. The arguments presented here are thus not specific to particular parameter values, but are generic for physically reasonable ranges of parameter values.

In the innermost region (denoted by 1), *a* ≤ *r* ≤ *r*_0_, the gradients are steep and the quadratic term can be neglected with respect to the derivatives. In this case, the solution to Eq. (16) can be approximated by ∇^2^*ϕ*(*r*) = 0 in 3D, so that *ϕ*_1_(*r*) = *a*^2^/(*rr*_0_), where the length scale *r*_0_ is determined from the mechanogen flux at *r* = *a*. Using this form *ϕ*_1_(*r*) for the concentration profile at the source *r* = *a* in the flux boundary condition of Eq. (8), we obtain 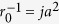, that is the length scale *r*_0_ scales as the inverse of the mechanogen flux.

In the outermost region (denoted by 3), *λ* ≤ *r* ≤ *L*, the linear degradation term dominates the quadratic term, and the mechanogen concentration is approximated by *ϕ*_3_(*r*) = *Aa*^2^*e*^−*r*/*λ*^/*r*. The pre-factor is determined from matching the solution in region 3 with an intermediate region 2 where the nonlinear degradation term dominates the linear one. In region 2, *r*_0_ ≤ *r* ≤ *λ*, the solution is expected to approximately obey the differential equation ∇^2^*ϕ*(*r*) − *a*^−2^*ϕ*^2^(*r*) = 0. In 3D, the entire set of analytic solutions of this non-linear equation is not known, although we can find one solution: *ϕ*_2_(*r*) = 2(*a*^2^/*r*^2^) which is an exact solution of the differential equation that is appropriate in this region. However, the coefficient determined in this manner may be crossover effect due to the lack of a large enough dynamic range of values of *a*, *r*_0_, *λ* and *R*. We determine the pre-factor *A* in region 3 by matching both the values and slopes of *ϕ*_2_(*r*) and *ϕ*_3_(*r*). These match at *r* = *λ* (where the solution of the nonlinear equation crosses over to that of the linear one where the mechanogen profile decays exponentially) and the pre-factor of the outer solution is determined to go as *A* ~ *λ*^−1^. This analysis is supported very well by Fig. S1 in the SI where we plot the exact numerical solution of Eq. (16) and show for comparison our analytic approximations in the three regions, which as the figure shows, match the numerical solution in the appropriate regions. The numerical solution of the differential equation shown in Fig. S1 is carried out for a representative set of parameter values (*r*_0_ = 50*a*, *λ* = 1000*a*, *R* = 20,000*a*) by shooting from the far *r* = *R* boundary with a value chosen to produce the necessary flux at the source and an appropriate relationship between the slope and value at *r* = *R* corresponding to a decaying exponential, as expected far from the source for a very large system. This case is numerically more tractable than one with reflecting boundary conditions, and the latter deviates appreciably from the decaying exponential only near the boundary.

We now use the approximate solutions in the different regions to calculate the average mechanogen concentration 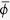 and then use the self-consistency condition of Eq. (7) for the 3D spherical geometry that, 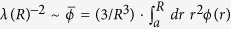, to find how *λ*(*R*) (that determines the exponential decay of the solution in region 3) scales with system size *R*. We perform this integral over each of the three regions defined above with the corresponding solution and find that the integral over region 1 scales as *r*_0_, while the integrals over regions 2 and 3 go as *λ*(*R*).


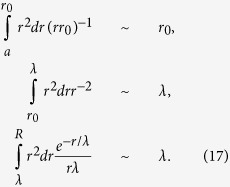


Keeping only the appropriate leading-order terms, the self-consistency condition suggests 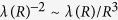, which predicts that *λ*(*R*) ~ *R*. This implies that the length that enters the exponential decay of the mechanogen concentration scales with the size of the system. For a system with clamped boundary conditions (*V*_0_ < 0), the mechanogen concentration decays exponentially to a finite bulk value 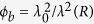 far from the source, which implies that the contribution to the total number of mechanogens from region 3 (third relation in Eq. (17) above) is now modified to 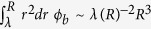, but leads to the same scaling 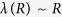 of the decay length of the mechanogen concentration with system size.

We explicitly demonstrate this size scaling of the concentration decay length by solving the self-consistent problem numerically for three different system sizes (with free mechanical boundary conditions)chosen to have the same value of source flux, using a shooting method as described above. The numerically obtained solutions for concentration, 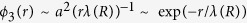, when plotted against the spatial coordinate rescaled by system size *nearly* collapse on each other in the far field exponential region, as shown in Fig. 2 in the main text. The corresponding decay length *λ*(*R*) values are obtained from the self-consistency condition 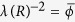 and also *nearly* scale with the values of *R* because the general dimensional analysis of the differential equation presented here is correct only in the asymptotic limit where *R* → ∞. The numerical results are never in the theoretically asymptotic limit and exhibit crossover effects that combine the solutions in regions 2 and 3. However, we see in Fig. 2 that the predicted scaling is obeyed more accurately for larger values of *λ*.

### Scaling for 2D circular geometry

We have presented in detail the results for the cases of a 1D line/ring of cells and a 3D spherical assembly. A geometry very pertinent to development corresponds to the blastula which is a hollow spherical shell of cells with a source of morphogens localized in a small region near the shell. This geometry can be mapped onto a 2D disk of cells with a mechanogen source in the region *r* < *a* and with free (zero stress) boundaries at the system edge at *r* = *R*. Strictly speaking, this mapping requires a blastula with two sources, one at each pole, but since these are local, the results should apply to the case of a single source. This gives rise to repulsive elastic interactions similar to the situation considered above in 3D. Proceeding as above (for a stress free boundary), we can show that the self-consistency condition leads to a similar linear size scaling of the concentration decay length with system size, up to logarithmic corrections. Similar to our discussion of the 3D case, the 2D solution can be approximately described by an inner *ϕ*_1_(*r*) = 1/(*rr*_0_), an intermediate 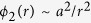, and an outer, 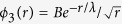 solution, where the matching condition yields *B* ~ *λ*^−3/2^. The self-consistency condition then suggests that *λ*^−2^ ~ *R*^−2^ log(*λ*/*r*_0_), or 
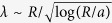
, which implies the decay length scales with system size in 2D up to weak logarithmic corrections. As in 3D, the case with clamped boundaries also predicts that the exponential decay of the mechanogen concentration scales with the size in the same manner as the free boundary case analyzed above, differing only in the value of mechanogen concentration far way (there is less degradation with clamped boundaries). 2D or 3D concentration profiles have longer ranges compared to the 1D case because the mechanogens, produced in a finite region, are relatively more dilute in 1D since there are more molecules in the outer regions in higher dimensions.

## Additional Information

**How to cite this article**: Dasbiswas, K. *et al*. Mechanobiological induction of long-range contractility by diffusing biomolecules and size scaling in cell assemblies. *Sci. Rep.* **6**, 27692; doi: 10.1038/srep27692 (2016).

## Supplementary Material

Supplementary Information
