# Supplementary material for: Mechanobiological induction of long-range contractility by diffusing biomolecules and size scaling in cell assemblies
Source: Sci Rep. 2016 Jun 10;6:27692. doi: 10.1038/srep27692 (PMC4901349; doi:10.1038/srep27692)
Supplement: Supplementary Information [file srep27692-s1.pdf]

Mechanobiological induction of long-range contractility by diffusing biomolecules and size scaling in cell assemblies
Supplementary Information

K. Dasbiswas *,[1] E. Alster,[2] and S. A. Safran[3]

[1] *James Franck Institute, University of Chicago, Chicago, IL 60637, USA & Department of Materials and Interfaces, Weizmann Institute of Science, Rehovot 76100, ISRAEL*

[2] *Department of Chemical Engineering, Northwestern University, Evanston, IL 60208, USA*

[3] *Department of Materials and Interfaces, Weizmann Institute of Science, Rehovot 76100, ISRAEL*

I. MECHANODIFFUSIVE COUPLING IN TERMS OF CONTRACTILITY

Here we show an alternative form of coupling of mechanogens that is equivalent in its results to the situation presented in the main text. Instead of supposing that the local cellular contractility, $\psi(\mathbf{x})$ is induced by, and therefore depends on the local mechanogen concentration alone, we additionally consider the effect on local contractility of the elastic interactions between cells, assumed to be mediated by their substrate (for model systems) or the ECM/direct cell-cell contacts (for embryos). Accounting for both effects allows us to write, in the spirit of linear response theory, an equation that relates the contractility of a cell at position $\mathbf{x}$ to both the *local*, effective, mechanogen concentration near the cell surface, $c(\mathbf{x})$, and the contractility of the other cells, as:

$$\psi(\mathbf{x}) = -\int d\mathbf{x}'\, \mathbf{V}(\mathbf{x},\mathbf{x}')\psi(\mathbf{x}') + \chi \mathbf{c}(\mathbf{x}), \tag{1}$$

where $V(\mathbf{x},\mathbf{x}')$ (which has units of inverse volume) accounts for the generally, long-ranged mechanical interactions of the contractile cells so that a cell at a position $\mathbf{x}'$ affects a cell at position $\mathbf{x}$ arbitrarily far away through the strain it induces in the underlying elastic medium. This accounts for the possibility that the local contractility is also actively changed by the cell in response to the local strain induced by the other contractile cells, such as through mechanically transduced myosin regulation[2]. This linear response of the local cellular contractility to both the mechanogen concentration and the contractility of the rest of the system can be motivated by considering the mechanical energy of the combined system of contractile cells and the mechanogens. We include in the energy terms the interaction of the cellular force dipoles[1] with each other as well as the binding of mechanogens to them as,

$$\mathcal{F}[\psi(\mathbf{x}), c(\mathbf{x},t)] = \int d\mathbf{x}\, \psi^{\mathbf{2}}(\mathbf{x}) + \int \mathbf{dx}'\, \psi(\mathbf{x}')\mathbf{V}(\mathbf{x},\mathbf{x}')\psi(\mathbf{x})\, \mathbf{dx} + \mathbf{F_c}[\psi(\mathbf{x}), \mathbf{c}(\mathbf{x},\mathbf{t})], \tag{2}$$

where $\psi(\mathbf{x})$ represents the local contractility (trace of the force dipole density tensor) and $c(\mathbf{x},t)$ the concentration of mechanogens; the energy is scaled by the energy cost of contractility in the absence of either other cells or mechanogens (the coefficient of the first term in Eq. (2)), and $F_c[\psi(\mathbf{x}), \mathbf{c}(\mathbf{x},\mathbf{t})] = -\chi \int \mathbf{dx}\, \mathbf{c}(\mathbf{x},\mathbf{t})\psi(\mathbf{x})$ represents the tendency of mechanogens to induce contractility with $\chi > 0$. Since mechanical forces are transmitted very quickly[3], the cell assembly can be considered to be in mechanical equilibrium and local binding equilibrium even if the mechanogens are not yet in their global, steady-state concentration profile. The contractility depends in an adiabatic manner on the local mechanogen concentration at a given time, $c(\mathbf{x},t)$, which evolves much more slowly than the dynamics of the mechanics. Therefore, minimizing the mechanical energy in Eq. (2) with respect to the contractility ψ, yields the condition for mechanical equilibrium, Eq. (1). However, this is only an example that motivates the linear response relation, Eq. (1), which is more general.

We close the mechanogen-contractility feedback loop by considering a scenario where the degradation of mechanogens is due to the cell contractility, (*i.e.*, the trace of the dipole tensor) which can happen through changes in their rate of uptake (via changes in membrane tension) or through cooperative binding of the mechanogens that show a preference to bind to contractile cells:

$$D\nabla^2 c - \beta\psi(\mathbf{x})c(\mathbf{x}) = 0 \quad \text{or} \quad \nabla^2 c - \lambda_0^{-2}\psi(\mathbf{x})c(\mathbf{x}) = 0. \tag{3}$$

---

* Correspondence to kinjald@uchicago.edu

This corresponds to case (ii)of strain-determined degradation considered in the main text, where the degradation couples to the local contractility instead of strain. Eqs. (1) and (3) together then provide a different, more formal, formulation of the mechanogen-contractility coupling that does not make any explicit reference to the strain induced by the dipoles, and are an alternative to the model described in the main text in terms of Eqs. (1) and (2). The effective equation we get for the mechanogen concentration after integrating out the contractility, $\psi(\mathbf{x})$ is equivalent to Eq. (4) in the main text, except for a reversal in the dependence on the mechanical boundary conditions, or in other words a change in the sign of the linear degradation term in Eq. (4). As we show in the main text, the scaling relations obtained for the decay length of the mechanogen profile with system size are not affected by this. Thus the size scaling is quite robust to the exact model or mechanism used for coupling mechanogen concentration to cell mechanics.

II. INTERACTIONS OF FORCE DIPOLES IN A FINITE MEDIUM

The theory of continuous elastic media determines that a distribution of forces induces stresses in the medium that satisfy a force balance[4] as,

$$\partial_j \sigma_{ij} = -f_i, \tag{4}$$

where σ_{ij} is the stress tensor (force per unit area), and $f_i(\mathbf{x})$ the local density of forces (per unit volume). In a linear elastic medium, the stress depends linearly on the strain through the elastic tensor,

$$\sigma_{ij} = C_{ijkl} u_{kl}, \tag{5}$$

where $u_{kl}(\mathbf{x}) = (1/2)(\partial_k u_l(\mathbf{x}) + \partial_l u_k(\mathbf{x}))$ is the strain tensor in terms of the local displacement $\vec{u}(\mathbf{x})$ of the medium.

We now relate the local force dipole density $p_{ij}(\mathbf{x})$ (dipole moment per unit volume) to the force density $\vec{f}(\mathbf{x})$. Force dipoles comprise two equal and opposite forces $\vec{F}$ that are separated by a distance $\vec{a}$. The product of the force and the distance defines a local force dipole[3]

$$P_{ij} = a_i F_j. \tag{6}$$

In a continuum representation (valid for scales much larger than $|a|$), one can consider the force density $\vec{f}(\mathbf{x})$ which is zero when integrated over some small volume Δ due to the equal and opposite forces of the dipoles. The force dipole is the first moment of the force density integrated over that volume.

$$P_{ij} = \int_\Delta a_i f_j(\mathbf{x}')\, d\vec{\mathbf{x}'}. \tag{7}$$

This relation can be inverted to give the local dipole density $p_{ij}(\mathbf{x})$. We consider a force distribution that comprises N pairs of equal and opposite forces separated by corresponding small distances (force dipoles). Writing this explicitly and expanding the expression for small distances, $\vec{a}$ between the forces we get

$$\vec{f} = \sum_{m=1}^{2N} \vec{F}_m\ \delta(\mathbf{x}-\mathbf{x}_m) = \sum_{m=1}^{N} \vec{F}_m\ (\delta(\mathbf{x}-(\mathbf{x}_m+\frac{1}{2}\vec{a}_m)) - \delta(\mathbf{x}-\mathbf{x}_m+\frac{1}{2}\vec{a}_m)) \approx \sum_{m=1}^{N} \vec{F}_m\ (-\vec{a}_m)\cdot\nabla\delta(\mathbf{x}-\mathbf{x}_m) \tag{8}$$

where we chose the signs of the δ functions so anti-parallel alignment of the vectors $\vec{a}$ and $\vec{F}$ result in a contractile force dipole. The force density 8 can then be written as a force dipole density,

$$\overleftrightarrow{p}(\mathbf{x}) = \sum_{m=1}^{N} \overleftrightarrow{P}_m \delta(\mathbf{x}-\mathbf{x}_m) \tag{9}$$

where $\overleftrightarrow{p}$ is the force dipole density tensor and the value of the local dipole tensor of the mth dipole is $\overleftrightarrow{P}_m = \vec{F}_m \vec{a}_m$. Taking the divergence of $\overleftrightarrow{p}(\mathbf{x})$ we get

$$\nabla\cdot\overleftrightarrow{p} = \sum_{m=1}^{N/2} \overleftrightarrow{P}_i \cdot\nabla\delta(\mathbf{x}-\mathbf{x}_m) = \sum_{m=1}^{N/2} \vec{F}_m \vec{a}_m \cdot\nabla\delta(\mathbf{x}-\mathbf{x}_m) = -\vec{f} \tag{10}$$

or

$$\frac{\partial p_{ij}(\mathbf{x})}{\partial r_j} = -f_i. \tag{11}$$

The work done by force dipoles to deform the medium is given by the product of the force applied by the dipole and the local displacement of the medium that is caused by that force, summed over the whole volume, as

$$W \equiv \int_V d\mathbf{x}\, f_i(\mathbf{x}) u_i(\mathbf{x}), \tag{12}$$

which using Eq. (11) can be written in terms of the force dipole density as,

$$W = -\int_V d\mathbf{x}\, \partial_j p_{ij}(\mathbf{x}) u_i(\mathbf{x}). \tag{13}$$

This after integrating by parts can be expressed as,

$$W = \int_V d\mathbf{x}\, p_{ij}(\mathbf{x}) u_{ij}(\mathbf{x}) - \int_S ds\, n_j p_{ij} u_j, \tag{14}$$

which has contributions from the dipole density in the bulk as well as from the surface of the finite domain, where $\hat{n}$ is the unit surface normal. This is expected as a distribution of force dipoles induces both volume and surface forces. (Here $\int_V$ implies an integral over the volume and $\int_S$ an area integral over the surface of the finite medium.)

The boundaries could be clamped or held fixed, such that the displacement is constrained to be zero at the surface, thus implying that the surface integral in Eq. (14) above vanishes. The other physically relevant condition is when the boundaries are free to move, or in other words are stress-free. This can be realized only if the surface forces from the dipoles in the medium are balanced by an externally imposed surface force distribution from the region outside the finite domain under consideration. Because the surface terms are zero in both of these two cases (Dirichlet and Neumann), the work done by a distribution of dipoles on the medium is the product of the dipole density with the local strain field[1,6],

$$W = \int_V d\mathbf{x}\, p_{ij}(\mathbf{x})\, u_{ij}(\mathbf{x}). \tag{15}$$

The central point of this Appendix is that this energy can be interpreted as the interaction of a dipole at a position $\mathbf{x}$ with the local strain field which is induced by all the *other* dipoles in the system[1,6].

III. ELASTIC DIPOLE INTERACTIONS IN A FINITE 1D DOMAIN

We now derive the mechanical interaction energy of a pair of force dipoles in 1D by considering an intuitive picture of a line of springs connected in series. A force dipole is represented as a single spring with a pair of equal and opposite forces (in this case compressive) applied on either end of it. We describe the discrete set of springs with a coarse-grained field $u(x)$ which characterizes the displacement of the springs at a position x. The ith spring is considered to lie between $x = (i-1)\cdot l$ and $x = i\cdot l$ where l is the length of each spring which is also the coarse-graining displacement in this description. We now apply this to a model of a finite medium, represented by a total of N springs thus forming a line of length $L = Nl$. The mechanical equilibrium condition relates the change of the deformation with a general discrete distribution of forces as[4],

$$kl\frac{d^2u}{dx^2} = -\sum_i F_i \delta(x - x_i), \tag{16}$$

where for a spring system, the 1D elastic modulus $E \equiv kl$, is more conveniently expressed in terms of the spring constant k (units of force per length). A single force applied at a point x_m therefore causes a jump discontinuity (seen by integrating Eq. (16)) in the slope of the displacement field, *i.e.* in the strain field,

$$\Delta\left(\frac{du}{dx}\right)|_{x=x_m} = -F/(kl), \tag{17}$$

This just implies that the springs to one side (right or left) of the force are compressed while those on the other side are stretched. A force dipole that compresses the mth spring therefore implies two equal and opposite jumps in the

slope of the displacement at positions $(m-1)\cdot l$ and $m\cdot l$ which causes all the springs to the left or right of the "dipole spring" to be deformed equally. There is no deformation of the other "external springs" if the ends of the line of springs are free to move since in that case the springs just translate. However, a nontrivial deformation can arise if the line of springs is held fixed or clamped at either end such that we require $u(0)=u(L)=0$. For a compressive force dipole that consists of a pair of forces, each of magnitude F, applied to either end of the mth spring, we find that each of the other springs in this line is stretched by a distance $F/(Nk)$ while the mth or "dipole" spring itself is compressed (change in length) by an amount F/k, thereby maintaining the constant overall length of the line. This solution satisfies the continuity of the displacement and the (two equal and opposite) jumps in its slope at $(m-1)\cdot l$ and $m\cdot l$ respectively as suggested by Eq. (17). The deformation of the dipole spring is much higher than those of the other springs representing the elastic medium (by a factor of N). In other words, if the dipole applies a pair of forces, each of magnitude F, the force in each of the other springs goes as F/N. The total energy stored in these springs then scales as $N\times(F/N)^2\sim 1/N$, *i.e.* inversely as the length of the line of springs.

Let us consider next two force dipoles applied at the mth and n^{th} springs where m and n are arbitrarily far apart. Let the forces of each dipole have magnitude F_1 and F_2 respectively. It can be shown by solving Eq. (16) with jumps in slope at positions m, $m+1$, n, and $n+1$ given by Eq. (17), that the deformation (change in length) of each of the "external" springs, *i.e.* all springs excluding the mth and n^{th} ones, is $(F_1+F_2)/(Nk)$. Summing over the deformation energies of all these springs yields the total energy of the line of springs with two dipoles. Subtracting from this the sum of the total energy in the line of springs for a single dipole at m and at n respectively, we obtain the energy of *interaction* of the two dipoles as,

$$W_{int}=-\frac{F_1F_2}{Nk}, \tag{18}$$

which is independent of the relative location of m and n, is negative, and depends inversely on the length of the line of springs. This intuitive description in terms of discrete springs thus confirms the form of interaction of force dipoles suggested by Eq.(4) in the main text. Note that we have used the work done to deform the springs rather than the energy stored in them as our definition of energy since this is the more relevant quantity for an active dipole[1].

IV. ELASTIC DIPOLE INTERACTIONS IN FINITE 3D SYSTEM

For an isotropic elastic medium, the condition for mechanical equilibrium for a distribution of forces can be written in terms of two elastic parameters as[4] (this is the higher dimensional analog of the 1D mechanical equilibrium expressed in Eq. (16)),

$$\mu\nabla^2\vec{u}+(K+\mu/3)\nabla(\nabla\cdot\vec{u})=-\vec{f}(\mathbf{x}), \tag{19}$$

where $\vec{u}(\mathbf{x})$ is the displacement of the medium at a position $\mathbf{x}$ induced by the local force density $\vec{f}(\mathbf{x})$, and K and μ are the usual bulk and shear modulus of the material.

If the forces arise from an isotropic distribution of spherical force dipoles $p_{ij}(r)=p(r)\delta_{ij}$, the forces produced by them and the consequent displacements in the medium are in the radial direction. In spherical polar coordinates, the dipole density tensor components are: $p_{rr}=p_{\theta\theta}=p_{\phi\phi}=p$, and all the other (off-diagonal)components are zero. In other words, the force dipoles have a trace(hydrostatic) component $3p(r)$ but no shear (deviatoric) components. Writing Eq. (19) in spherical polar coordinates[4,5], we obtain,

$$\frac{d^2u}{dr^2}+\frac{2}{r}\frac{du}{dr}-\frac{2u}{r^2}=(K+\frac{4}{3}\mu)^{-1}\frac{dp}{dr}, \tag{20}$$

where we have used the relation between force and dipole density, $f(r)=-dp_{rr}/dr=-dp/dr$, obtained in Eq. (11). The left hand side of Eq. (20) can be expressed as,

$$\frac{d}{dr}\left(\frac{1}{r^2}\frac{d}{dr}\big(r^2u(r)\big)\right)=\frac{p'(r)}{(K+\frac{4}{3}\mu)}, \tag{21}$$

where the prime refers to differentiation with respect to r. Integrating this expression twice in succession, we obtain the displacement as,

$$(K+\frac{4}{3}\mu)\frac{1}{r^2}\frac{d}{dr}\big(r^2u(r)\big)=p(r)+c_1/3, \quad \text{or} \quad (K+\frac{4}{3}\mu)u(r)=\frac{1}{r^2}\int dr\, r^2p(r)+c_1r+\frac{c_2}{r^2}, \tag{22}$$

where the integral is indefinite, and c_1 and c_2 are integration constants determined by suitable boundary conditions. Since symmetry requires that the system is fixed at the center $r = 0$, c_1 is determined such that the radial displacement can be written as,

$$u(r) = (K + \frac{4}{3}\mu)^{-1}\left[\frac{1}{r^2}\int_0^r dt\, t^2 p(t) + c_1 r\right], \tag{23}$$

while the corresponding expression for the radial component of the local strain is,

$$u_{rr} \equiv \frac{du}{dr} = (K + \frac{4}{3}\mu)^{-1}\left[\frac{-2}{r^3}\int_0^r dt\, t^2 p(t) + p(r) + c_1\right] \tag{24}$$

We consider this solution in a sphere of finite size R (corresponding to a finite spherical assembly of cells). The constant c_2 is determined from the boundary conditions at the surface of the sphere. If the sphere is clamped at the surface, *i.e.*, $u(R) = 0$, the corresponding displacement can be written as,

$$(K + \frac{4}{3}\mu)u_{clamp}(r) = \frac{1}{r^2}\int_0^r dt\, t^2 p(t) - \frac{r}{R^3}\int_0^R dt\, t^2 p(t), \tag{25}$$

while absence of radial stresses at the surface (free boundary conditions) leads to a displacement,

$$(K + \frac{4}{3}\mu)u_{free}(r) = \frac{1}{r^2}\int_0^r dt\, t^2 p(t) + \frac{2r}{R^3}\int_0^R dt\, t^2 p(t) - rp(R), \tag{26}$$

The trace of the strain(in radial coordinates)[4] defined as $Tr(u) \equiv du/dr + 2u/r$, for clamped boundaries is then,

$$Tr[u_{ij}^{clamp}(r)] = \frac{1}{K + \frac{4}{3}\mu}\left(p(r) - \frac{3}{R^3}\int_0^R dt\, t^2 p(t)\right) \equiv \frac{p(r) - \overline{p}}{K + \frac{4}{3}\mu}, \tag{27}$$

and for free boundaries,

$$Tr[u_{ij}^{free}(r)] = \frac{1}{K + \frac{4}{3}\mu}\left(p(r) - \frac{6}{R^3}\int_0^R dt\, t^2 p(t)\right) \equiv \frac{p(r) + 2\overline{p}}{K + \frac{4}{3}\mu}, \tag{28}$$

where $\overline{p}$ is the volume average of the isotropic dipole density, and for the free boundaries, we have dropped the constant $p(R)$ which would anyway not contribute to the elastic interaction energy. Exactly analogous to the 1D case, the local strain in the medium thus has two contributions: one from the local dipole density and the other being a sum of the contributions of all the other dipoles in the finite medium. Note that we use ψ to represent the nondimensional isotropic cell contractility in the main text; which is related to the force dipole density, here denoted by p as $\psi(x) = p(x)/p_{max}$ p_{max} is the maximum contractility of a cell and p is negative by convention for contractile dipoles. The local dipole is always contractile and thus contributes a compressive strain irrespective of the boundary conditions. The other dipoles stretch the medium when the boundaries are fixed while they compress the medium if the boundaries are free. This is reflected in the change in sign of the second term between Eqs. (27) and (28).

The mechanical energy invested by force dipoles in deforming the medium was derived in Appendix II to be the product of the local dipole density and the local strain field produced by all the other dipoles, which is represented by the term $-\overline{p}/(K + (4/3)\mu)$ in Eqs. (27) and Eqs. (28):

$$W = \int dr\, r^2 \int dr'\, r'^2 p(r)V(r, r')p(r') \tag{29}$$

which implies that the interaction kernel $V(r, r')$ between an isotropic dipole at $\vec{r}$ and one at $\vec{r'}$ has the simple constant form for clamped boundaries,

$$V_{clamp}(r, r') = -\frac{3}{R^3}(K + \frac{4}{3}\mu)^{-1} \tag{30}$$

which is negative (representing an attractive interaction). The corresponding interaction for free boundaries is,

$$V_{free}(r, r') = \frac{6}{R^3}(K + \frac{4}{3}\mu)^{-1} \tag{31}$$

which is positive (representing a repulsive interaction). Both the clamped and free boundary conditions lead to pairwise interactions between dipoles that are independent of the distance of separation between dipoles, and are inversely related to the system volume. They come with opposite signs and differ in strength by a factor of 2 but have the same generic behavior as seen above. The inverse volume scaling is responsible for the relation in Eq. (5) in the main text, whereas the sign of V_0 in Eq. (4) in the main text is determined according to the mechanical boundary condition by Eq. (30) and (31) above.

Physically this kernel V(r,r') represents the interaction between a dipole and the images of the other dipoles[6], since there are no direct elastic interactions between isotropic dipoles in an infinite medium. For a more general, anisotropic distribution of dipoles, the elastic interactions of dipoles in a finite 3D sphere can be expanded in the basis of spherical harmonics as[7],

$$V(\mathbf{x},\mathbf{x}') = \frac{1}{R^3}\sum_{lm} M_l \left(\frac{r}{R}\right)^l Y_{lm}(\Omega)\left(\frac{r'}{R}\right)^l Y_{lm}(\Omega'), \quad (32)$$

where M_l is negative (positive) if the boundaries are clamped (free), and scales inversely as the system volume. The magnitude of M_l decreases monotonically with l and we see from the form of Eq. (32) that the zeroth order contribution is dominant especially for dipoles far from the boundaries of the sphere. This justifies our consideration of an isotropic distribution of dipoles in this paper while also showing that higher order interactions are long-ranged and scale similarly with system volume; they are thus expected to give the same scaling relations for the mechanogen gradient vs. system size. Finally, we remark that since the mechanogen tensor is a scalar, it couples directly only to the trace of the strain or dipole tensors.

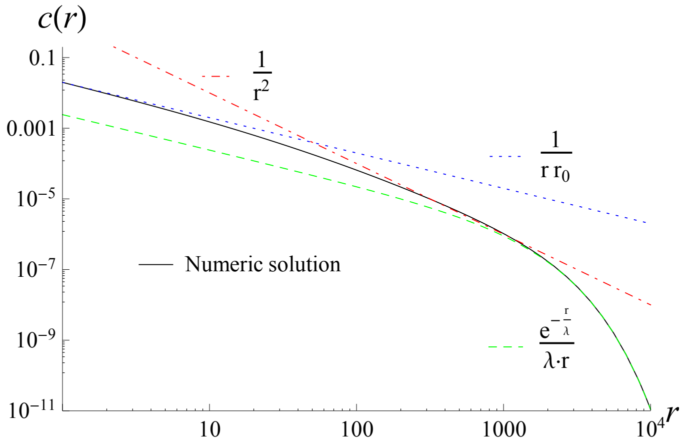

FIG. 1: **Comparison of numerical solutions for the 3D geometry with approximate analytical solutions in different regions.** The solution of Eq. (**??**) presented and analyzed here is appropriate when the different relevant length-scales are well-separated: all other length scales are defined in terms of a, the size of the source. These are: the length scale characterizing the flux. $r_0 \sim j^{-1} = 60a$, the linear decay length $\lambda(R) = 1000a$, and the size of the domain over which the diffusion-degradation Eq. (4) in the main text is solved, $R = 20,000a$, which is practically infinite. We show several simple analytic expressions for different regions of r and compare them to the numerical solution. In the innermost region where $r \leq r_0$ we approximate $\phi_1(r) = a^2/(rr_0)$ (dotted, blue line); in an intermediate region with $\phi_2 = a^2/r^2$ (dashed-dotted, red line), and in the outer region where r , $\phi_3(r) = a^2 \exp[-(r-\lambda)/\lambda]/(r\lambda)$ (green, dashed line). The pre-factor (e/λ) is determined by matching the outer and the intermediate solutions. While $\phi_2(r) = 2a^2/r^2$ exactly solves Eq. (4) in 3D with only the gradient and quadratic terms (which are the dominant ones when the concentration is relatively large), in practice we see an intermediate region which better fits a pre-factor of unity, namely $\phi_2 = a^2/r^2$. The difference of a factor of two in the numerical pre-factor is probably a "crossover effect" arising from having to match the outer exponential solution with the inner power law behavior over numerical values of r that do not give a good enough separation of length scales. We have verified numerically that the pre-factor of the $1/r^2$ term tends to increase from 1 to 2 as the separation of the scales r_0 and $\lambda(R)$ is increased, thereby providing further evidence that these are crossover effects. (Color online)

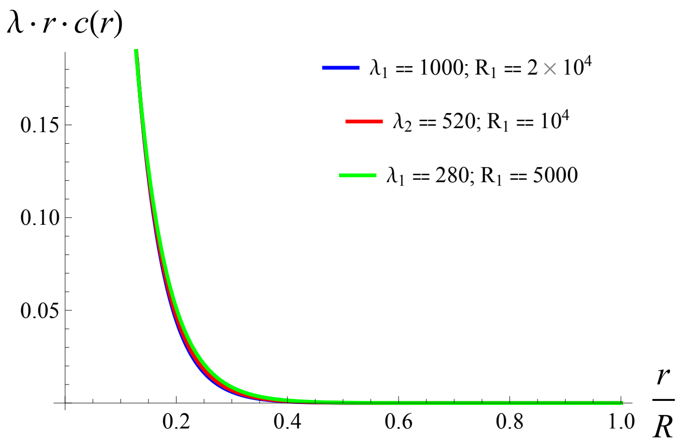

FIG. 2: **Self-consistent solutions in 3D vs. radial coordinate rescaled with system size, R, showing approximate scaling:linear plots** We solve the diffusion-degradation equation in 3D self-consistently, numerically for three different system sizes $R_1 = 20,000a$ (blue), $R_2 = 10,000a$ (red) and $R_3 = 5000a$ (green)and find the corresponding decay lengths $\lambda(R)$= $1000a$, $520a$ and $280a$ respectively from the self-consistency condition $\lambda(R) \sim \sqrt{\phi}$ stated in the main text. This is a linear plot of the same solutions plotted on a semilog scale in Fig. (2) of the main text and exhibits approximate scaling as reported there. (Color online)

[1] Schwarz, U. S. & Safran, S. A., Physics of adherent cells. Rev. Mod. Phys. **85**, 1327 (2013).
[2] Kovács, M., Thirumurugan, K., Knight, P. J. & Sellers, J. R. Load-dependent mechanism of nonmuscle myosin 2. Proc. Natl. Acad. Sci. USA **104**(24): 9994–9999 (2007).
[3] Yuval, J. & Safran, S. A., Dynamics of elastic interactions in soft and biological matter. Phys. Rev. E **87**, 042703 (2013).
[4] L. D. Landau and E. M. Lifshitz, Theory of Elasticity (Pergamon, Oxford, 1970), 2nd ed.
[5] A. Bower, Applied Mechanics of Solids, CCRC Press, Boca Raton, FL, USA, (2009).
[6] Bischofs, I. B., Safran, S. A. & Schwarz, U. S. Elastic interactions of active cells with soft materials. Phys. Rev. E **69**, 021911(2004).
[7] Wagner, H. & Horner, H., Elastic interaction and the phase transition in coherent metal-hydrogen systems. Adv. Phys.**23**, 587 (1974).
